# Philo-Medicus, on Medical Graduation

**Published:** 1802-04

**Authors:** 


### Philo-Medicus, *on Medical Graduation*.

To the Editors of the Medical and Physical Journal.

Gentlemen,

I Have read with pleasure in your last Number, the answer of an Old Practitioner, and the remarks of Aristarchus, jun. The former alleges, I am "a *young physician* who wishes well to the character, but who knows too little of the *character*, and is somewhat too fast." I can assure my old friend, that *I wish well to the character*, but am only a *spectator*. I have neither an Edinburgh, Glasgow, nor St. Andrew's Degree, nor one from any where else; hence there need be no suspicions that what I have said is out of envy at any part of the Profession, because they are not "of the same College." I can assure him too, I am as much a friend to real worth as he can be. I am of the same opinion with him, that "it would be better for the world if *sterling merit* were more prized than NOMINAL HONOURS." That "a Degree will never make a Physician," is the very point I am wishing to establish. A Degree is the Certificate of being qualified, when properly obtained; but not the Qualification.

"Never tell me," says your Correspondent, "of the superlative honour of certain Degrees, until Professors shall require of every Candidate to give oath that he did not attend those gentlemen called *Grinders*." Is not a *Grinder* a different name for a private Teacher? If so, what is the difference, whether a man be instructed in public, in private, or in both, provided he acquire sufficient knowledge before he obtain his Degree? I appeal to the old Gentleman himself, if it be not better to attend for "three years and nine months," and during that time to obtain knowledge by public teaching, grinding, and all the other means usually employed [illegible] preparing students, and then to receive a Degree? Or, to begin by first practising for a few years, and after we have acquired a little knowledge at the expence of the lives of our fellow-creatures, to think of taking out a St.

a St. ——'s Degree, to make us refpectable Phyficians? I hope for the honour of humanity, there are few of this opinion. If a man by the little learning he has got, by his common fenfe, and by carefully obferving the operations of Nature, has in the courfe of a few years made himfelf a refpectable practitioner, what greater honour can he expect from having a St. ———'s Degree? The principal thing neceffary to the procuring of which, is to find a man who will carry the money and bring back the Degree. Thus, in place of *Grinders*, we find *Mail-Coachmen* and *Carriers* neceffary aids towards Graduation.*

All the Gentlemen who have Degrees from the Colleges of St. ——— and A——, are not equally liable to thefe cenfures; there are fome very refpectable characters, who have practifed for many years, and who have had recourfe to this method: They are, however, to blame; for although they may be fufficiently qualified, it is leaving an open door through which perfons of a very different defcription may pafs. I confider my friend, from what he has faid, to be a perfon fully qualified to practife as a Phyfician, hence there was no impropriety in giving *him* a Degree: But what would he think, inftead of being affociated with "an active, fteady young man, who has ferved an apprenticefhip of five or fix years, and who has feen a good practice during that time," to be affociated with a lazy, felf-conceited weaver, who has left his loom, ferved an apprenticefhip of only a year or two with a man little better qualified than himfelf, and inftead of being at College "three years and nine months," only *four* or *five* months, and afterwards to practife till he could mufter up as much money as would purchafe a Degree? Such characters, however odious they may appear, are to be found among my friends, Fellow Phyficians!

The old Gentleman fays, "he glories to tell that he has long practifed under the banner of a Degree from the ancient and honourable College of St. ———." I believe him well; but men who do not reflect on the caufes and confequences of their actions, often "glory in their fhame." The caufe of his taking a St. ———'s Degree he has not mentioned; but the confequences are evident. I fuppofe my friend to be a Gentleman of refpectability, and one who really does honour to the profeffion. The world knows his Degree is from St. ———. Another man, who would be a difgrace to *any* profeffion, by fome means or other, procures a Certificate, and obtains

* "Ridicule," fays Dr. Blair, "will often fucceed in reforming Vice and correcting Error, when Reafon and Arguments have failed."

obtains a Degree. Now he is upon a footing with my friend, and the public as naturally confide in the one as in the other, fince they are "both practifing under the banners of Degrees from an ancient and honourable College!"

But if individuals are to blame for procuring fuch Degrees, what culpability are we to attach to a body of men who thus proftitute the honours of an *ancient and honourable College?* They may plead their poverty; but a man's poverty is feldom a fufficient excufe for deviating fo far from the path of rectitude. This my good old friend will call *railing*; but he fhould remember the proverb, *Desperate diseases require desperate remedies.* Had I not ufed ftrong language, my communication might not have been taken notice of, and the end would have been loft. But now, fince two champions have taken the field, there is little doubt of fetting every thing in its proper light.

Ariftarchus wifhes to know if I have been refufed *literary honours.* I fee no reafon for afking this queftion, and as little for anfwering it. To him it is certainly a matter of indifference whether I am phyfician, furgeon, apothecary, or nothing, fince the "Animadverfions of every medical *controvertist* fhould be directed to the *subject matter* only, without any regard to the *authors* of opinions in difpute." I have been charged with ufing harfh language, but this is merely an opinion. If the fubject required it, harfh language became neceffary; and my ftyle would not have been appropriate had I not ufed it. What one gentleman confiders as harfh, illiberal, and unbecoming, another declares to be "laudable, well timed, and ingenuous."* So there is no deciding in matters of this kind; every man fpeaks as he *feels.*

I am of opinion with you, that Controverfy without *salt* will foon become infipid.† No man would think of writing upon a fubject of controverfy, or on one fo notorioufly hurtful to fociety, as that of granting Degrees to perfons unqualified,‡ in the fame ftyle as he would write a love epiftle. I could produce many inftances where the moft beneficial effects have refulted from controverfy, when it was carried on in the moft perfonal manner, but I fhall fatisfy myfelf with one or two. The Difputes which happened between Dr. Cullen and Dr. Brown, and between Dr. Hunter and Dr. Monro, were carried

---

* Medical and Phyfical Journal, vol. vii. p. 281.

† Note, vol. vii. p. 261.

‡ Whatever may have been the practice formerly, we have reafon to believe that at prefent they do not grant Degrees without due fatisfaction refpecting the qualifications and character of the recommenders, as well as the reommended. EDIT.

ried on with the utmoſt invective; yet, notwithſtanding, theſe controverſies gave riſe to theories and diſcuſſions which have done much to the improvement of medicine. I believe that many of the ingenious theories of theſe gentlemen were ſtarted more with a view to thwart their antagoniſts, than to improve the healing art. But be this as it may, they mutually gave riſe to more philoſophical reſearch than was formerly known in the medical world. The Letters of Junius were full of invective and perſonalities; but will any man diſpute but they were of much advantage to the country at the time they were written? Public grievances and abuſes are not to be removed by a ſervile attention to all the formalities of faſhion, but by bold detection and expoſure to the world.

There is nothing more detrimental to the advancement of ſcience than compleatly following cuſtoms and authorities. To doubt is the firſt ſtep towards improvement. For many hundred years the world was fettered by the cuſtoms and doctrines of the ancients; but no ſooner did men begin *to doubt*, than Knowledge ſpread like lightning over the Univerſe. I hold it neceſſary to the advancement of ſcience that there ſhould be a diverſity of opinions. Nothing is a greater ſtimulus to exertion than a perſonal diſpute. It ſhews that a man's opinions are taken notice of; and what was ſaid of the Iſraelites in Egypt may be ſaid of them, the more they are oppreſſed the more they multiply. The minds of the individuals themſelves are often warped with prejudice, but the cool ſpectator never fails to catch a ſpark of knowledge from the colliſion of jarring parties. I have read many diſputes in your valuable Journal; in ſome of them, things were ſaid which might well have been omitted, but from all I derived improvement.

By giving the preceding reflections a place in your Journal you will perhaps contribute to the removal of an abuſe which has long been complained of, and you will oblige,

Your's, &c.

*Scotland,*
*March* 9, 1802.

PHILO-MEDICUS.

